# Improving Affective Associations With Physical Activity via a Message-Based mHealth Intervention (WalkToJoy): Proof-of-Concept Study

**DOI:** 10.2196/75792

**Published:** 2025-08-08

**Authors:** Soo Ji Serisse Choi, Pei-Yao Hung, Mengyun Liu, Walter Dempsey, Mark W Newman, Predrag Klasnja

**Affiliations:** 1 School of Information University of Michigan Ann Arbor, MI United States; 2 Institute for Social Research University of Michigan Ann Arbor, MI United States; 3 Department of Biostatistics University of Michigan Ann Arbor, MI United States

**Keywords:** mobile health, affective messaging, physical activity promotion, older adults, affective-reflective theory, evaluative conditioning

## Abstract

**Background:**

Traditional mobile health interventions for physical activity (PA) primarily rely on reflective self-regulatory processes, often neglecting the role of affective associations in sustaining long-term engagement. The WalkToJoy intervention addresses this gap by applying the affective-reflective theory to enhance intrinsic motivation for PA among adults aged ≥40 years through affective message framing, evaluative conditioning, and belief updating.

**Objective:**

This proof-of-concept study evaluated the feasibility of the message-based WalkToJoy intervention package and examined the impact of its 3 components—walking suggestion prompts, salience messages, and planning prompts—on affective and behavioral outcomes related to walking.

**Methods:**

We conducted a fully remote, 6-week full factorial experiment with an embedded microrandomized trial (MRT) involving 49 adults aged ≥40 years. Statistical analyses, including paired *t* tests and generalized estimating equations, assessed pretest-posttest changes and the effects of smile-inducing walking suggestion prompts with short animated images (GIF images), salience messages, and planning prompts on weekly affective measures and daily step counts. In addition, MRT analyses evaluated the proximal effects of these components. Poststudy interviews were thematically analyzed to contextualize participants’ experiences and engagement with the intervention.

**Results:**

Significant pretest-posttest improvements were observed across affective outcomes on a 7-point Likert scale—affective attitudes improved by 0.547 points (*P*<.001), affective valuations improved by 0.718 points (*P*<.001), affective reflection improved by 0.692 points (*P*<.001), and anticipated affect improved by 0.692 points (*P*<.001). While the average daily steps showed a nonsignificant pretest-posttest increase of 80 steps (*P*=.79), further analysis revealed an increase of 506 steps (*P*=.07) when comparing baseline to the average of weeks 4 to 6. Among the intervention components, GIF prompts significantly increased anticipated affect by 0.345 points (*P*=.046) and average daily step count by 1834 steps (*P*=.05) compared to identical text-only prompts. However, MRT analysis found no significant increase in 4-hour step counts following the walking suggestion prompts (*P*=.55), which was explained by qualitative findings suggesting that participants interpreted messages as flexible day-long reminders rather than immediate calls to action. Salience and planning prompts did not yield substantial quantitative effects but were positively received by participants for promoting mindfulness and personalized engagement.

**Conclusions:**

The WalkToJoy intervention is a feasible and promising approach for improving affective associations with walking. Walking suggestion prompts were particularly effective in boosting engagement and mitigating message fatigue, highlighting the potential of affect-driven interventions to enhance PA motivation and adherence.

## Introduction

### Background

Physical activity (PA) promotes long-term health and wellness by providing physiological benefits such as weight management, blood glucose and blood pressure control, and reduction of the risk of chronic diseases such as heart disease and diabetes. In addition, it enhances cognitive functions (eg, improved alertness, attention, and memory) [1-3] and emotional well-being (eg, elevated mood and confidence and reduced anxiety and stress) [1,3,4]. Despite the many documented benefits of PA for health and wellness, increasing PA at a population level remains a challenge. Research shows that, in 2020, only 24.2% of American adults met the recommended levels of PA (ie, 150 minutes of moderate-intensity PA and 2 days of muscle-strengthening activity per week) [5]. In addition, PA levels decline significantly with age, particularly after midlife, contributing to the heightened prevalence of cardiometabolic risks—including hypertension and insulin resistance—among older adults [5]. With an aging population and extended life expectancy, promoting sustained PA in middle-aged and older adults represents an increasingly significant public health challenge.

With a high penetration of smartphones and wearables across a broad range of populations, there have been many interventions that have attempted to provide support for PA through mobile health (mHealth) apps [6]. Compared to the more traditional modes of intervention, such as in-person cognitive behavioral therapy, the key benefits of mHealth apps are high accessibility, low cost, support for user autonomy [7-9], trackability of progress over time with continued intervention support in daily settings, and the potential for adaptation and tailoring to individual needs [10-12]. However, despite these advantages, mHealth interventions continue to face persistent challenges in sustaining long-term engagement, particularly among middle-aged and older adults [13,14]. In this population, psychological barriers—such as low intrinsic motivation, reduced self-efficacy, fear of injury, negative age stereotypes, and previous negative experiences with PA [13-16]—are often compounded with age-related physical limitations (eg, chronic conditions and fatigue) and technological barriers (eg, lower digital literacy and poor usability for older adults) [17,18]. These overlapping challenges can create a self-reinforcing cycle of disengagement. To break this cycle, sustaining long-term engagement among older adults requires a fundamentally different design approach—one that is more resilient to age-related changes, fluctuating motivation, and psychological barriers.

### Traditional Theory-Based mHealth Interventions and Their Limitations

Scholars in the mHealth space have relied on behavior change theories and models such as self-determination theory, the Health Belief Model, the theory of planned behavior, and social cognitive theory to understand the determinants of health behaviors and guide the design of intervention strategies. These theories provide valuable insights into the psychological factors that drive intention and motivation for behavior change, such as perceived benefits, self-efficacy, and autonomy. By identifying the *why* behind behavior, these frameworks have informed the development of theory-based strategies to create mHealth interventions tailored to psychological and behavioral needs.

Despite the theoretical rigor of traditional behavior change models, their heavy reliance on self-regulatory strategies such as goal setting and planning assumes that health behaviors are primarily driven by reflective cognitive processes. However, growing evidence highlights the significant role of subconscious and automatic processes, including implicit attitudes, emotions, and habits, in shaping behavior [19-21]. These implicit mechanisms operate below conscious awareness, shaping perceptions and actions through repeated experiences and associations [22]. In recognition of these limitations, health psychology has increasingly shifted toward dual-process theories, which integrate both reflective and automatic processes to better capture the complexity of behavior change. This shift is particularly relevant for PA adherence, where motivation type plays a critical role. Research shows that individuals driven by intrinsic motivation (eg, enjoyment) sustain activity longer than those driven by external motives (eg, appearance or peer approval) [23,24]. Affective states—feelings of pleasure or displeasure—are central to intrinsic motivation, shaping behavioral choices through subconscious affective processing [23,25-27]. Fostering positive affective experiences with PA has proven especially promising for encouraging sustained engagement, particularly among sedentary individuals [27].

### Intervening on Affective Determinants of PA

The affective-reflective theory (ART) of physical inactivity and exercise is a dual-process framework for understanding how both automatic and reflective processes shape exercise behavior. Central to ART is the concept of affective valuations—momentary emotional responses to exercise behavior based on previous experiences—which arise automatically with minimal cognitive effort [20,28]. These fast, automatic responses interact with slower, reflective processes that involve conscious reasoning through working memory [20,28-30]. According to ART, repeated experiences with PA form affective valuations—instant reactions of attraction or aversion triggered by thinking about or preparing to engage in exercise. Over time, these valuations, reinforced through reflection on one’s experiences, develop into affective attitudes—more stable emotional evaluations of the behavior (eg, likes or dislikes) [22]. Together, affective valuations and attitudes—referred to collectively as affective associations—play a critical role in shaping one’s behavior, performance, and exercise decision-making [30-34]. Therefore, sustaining positive affective associations (eg, enjoying PA) is essential for strengthening intrinsic motivation and intention to engage in regular exercise [20,28,29].

However, despite the close relationship between affective associations and the motivation and intention to engage in PA, mHealth interventions largely overlook this critical determinant of PA. Recent mHealth interventions have attempted to track and monitor affective states, but most of them fall short of directly influencing the core affective association processes that underpin the intrinsic motivation for engaging in PA [30,31,35]. While a few recent studies have shown promising results in modifying implicit attitudes within controlled laboratory settings [36-38], this approach has not been widely tested in mHealth interventions that support users in the dynamic, real-world contexts of everyday life. One notable exception is HeartPhone [39], an mHealth intervention that presented emotionally positive images of people exercising on users’ smartphone lock screens over an 8-week period, resulting in improved affective judgments (ie, enjoyment, intrinsic motivation, and integrated regulation).

Although the research that specifically focuses on improving affective associations with PA is still relatively nascent, there is extensive literature on messaging that indicates that message framing can effectively impact motivation for healthy behaviors such as PA [40-43]. Affective content can play an important role in such messaging interventions, supporting the idea that messaging can engage automatic affective processing and elicit subconscious emotional responses. For instance, a study using affective message framings demonstrated promising results in influencing exercise behavior and shifting attitudes toward PA [44]. Previous research has also shown that emotional imagery enhances affective arousal, valence, and attention [45,46], suggesting that images might be a powerful way of actuating affective processes in messaging interventions.

How best to leverage these insights in mHealth interventions to effectively influence affective processes toward PA, particularly among middle-aged to older adults, remains an open research challenge. To begin to address this gap, this paper presents a proof-of-concept study of the WalkToJoy mHealth intervention. Grounded in ART, this message-based mHealth intervention was designed to improve middle-aged and older adults’ affective valuations of and affective attitude toward walking (ie, a moderate PA) to support their ability to sustain PA in the long term.

### Theoretical Underpinnings of the WalkToJoy Intervention

**Figure 1:**
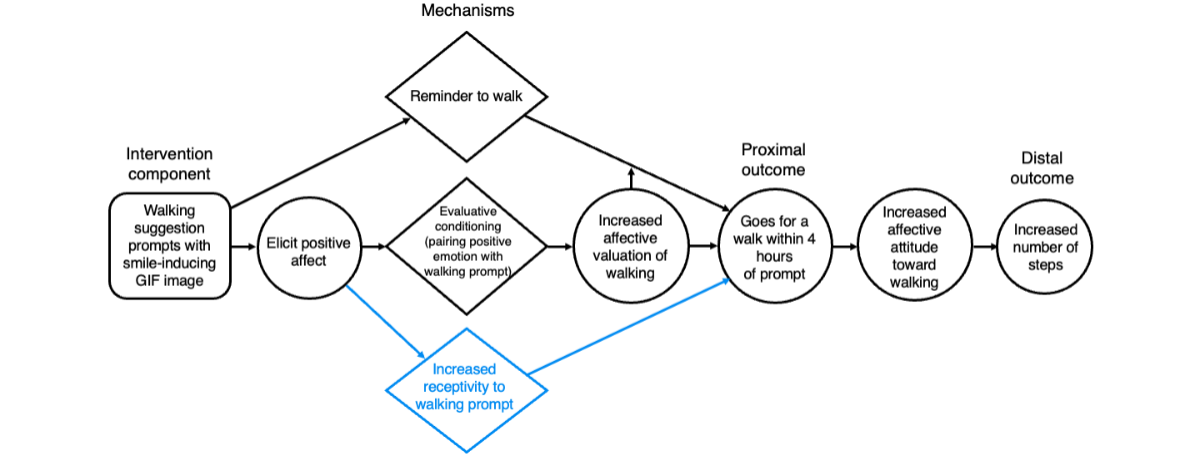
Causal pathway diagram of evaluative conditioning via walking suggestions. The intervention was designed to work through reminding and evaluative conditioning (2 top pathways). Our results suggested that a third mechanism, increased receptivity (shown in blue), operated as well.

WalkToJoy was designed to be a low-cost, scalable intervention that can augment the standard reflective self-regulatory strategies, such as self-monitoring and goal setting, found in commercial activity trackers (Fitbit in this case) with SMS text messages aimed at improving middle-aged and older adults affective associations with PA. Theoretically, the WalkToJoy messages implemented 2 key strategies to improve affective associations: evaluative conditioning [37,38] and belief updating [47]. Evaluative conditioning [37,38] involves repeatedly pairing an inherently positive (for promotion) or negative (for cessation) stimulus with representations of the behavior one is trying to impact. WalkToJoy used evaluative conditioning by pairing a PA-related stimulus (ie, a walking suggestion) with smile-inducing animated images of cute animals in GIF images to subconsciously enhance users’ affective valuations of walking. Unlike HeartPhone [39], which facilitated evaluative conditioning of PA by showing attractive images of people being active on the lock screens of users’ phones, WalkToJoy implements evaluative conditioning by embedding positively valenced imagery within *push* notifications that prompt walking. This design embeds evaluative conditioning into a behavioral cue, enhancing its potential impact on walking behavior. We hypothesized that repeatedly pairing, via push messages, suggestions to go for a walk with an intrinsically positive emotional experience—smiling induced via a GIF image of a cute animal—would, over time, strengthen associations between walking and positive emotions, resulting in increased walking behavior as a distal outcome (Figure 1).

**Figure 2:**
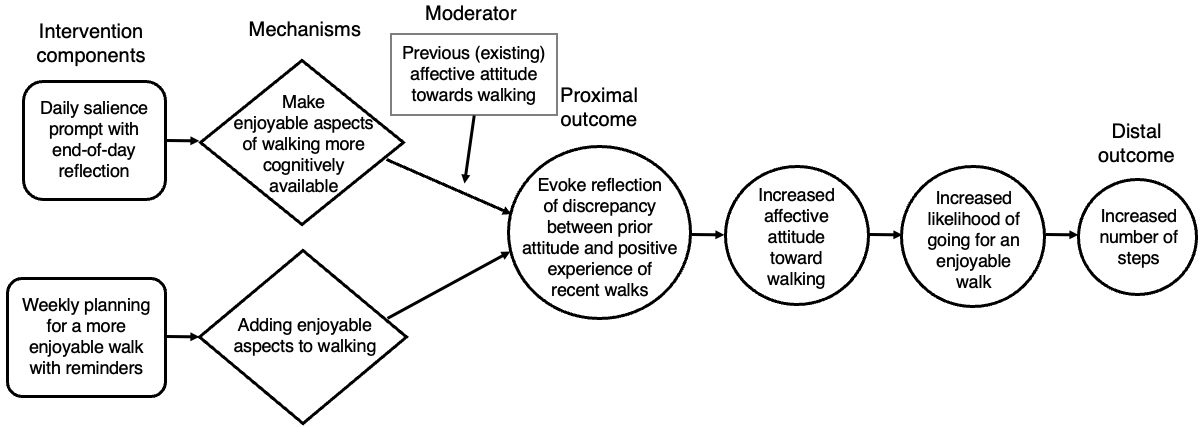
Causal pathway diagram of belief updating via salience and planning.

Belief updating is a strategy that aims to reshape individuals’ expectations and previous beliefs about a behavior [47]. According to ART, beliefs about PA—including affective attitudes—can be reshaped by prompting individuals to notice and reflect on the positive experience of being active [48-50]. Previous studies have emphasized the effectiveness of positively framed messages and action planning on aging adults’ PA [41,51-53]. WalkToJoy builds on this research by specifically targeting belief updating by making enjoyable aspects of walking more cognitively accessible and by encouraging PA exploration by planning more enjoyable walking experiences. Mechanistically, these components were intended to evoke reflection on the discrepancy between the positive aspects of walking, which planning and salience components supported, and one’s previous attitudes toward walking (Figure 2). The intended proximal outcomes were for participants to report greater enjoyment and anticipation of their daily walks, which, over time, would enhance affective attitudes toward walking and increase the likelihood of engaging in PA (distal outcome).

To assess the feasibility of this intervention and gather initial evidence for its mechanistic impacts, we conducted a 6-week proof-of-concept study of WalkToJoy. This study was conducted within the Multiphase Optimization Strategy framework [54], and it used a hybrid experimental design that combined a factorial experiment with a microrandomized trial (MRT) to investigate the potential of each of WalkToJoy’s 3 experimental components to influence affective associations and walking behavior. In addition, we interviewed a subset of participants to understand their experiences with the intervention and how they incorporated walking into their daily lives.

## Methods

### Overview

A proof-of-concept study of the WalkToJoy intervention was conducted as a 6-week full factorial experiment with 3 factors (ie, for a total of 8 conditions) and an embedded MRT [11,13,41,55]. The primary aim of this study was to assess the feasibility of the WalkToJoy intervention package as a whole for improving affective associations with walking. The secondary aim was to assess how each of the 3 components contributed to an improvement in affective associations with walking and the walking behavior itself. In addition, this study investigated the impact of the microrandomized components on their corresponding proximal outcomes to inform the decision rules (eg, timing and context) for the delivery of those components. This study was retrospectively registered with the Open Science Framework [56] after data collection and analysis were completed.

### Recruitment

The target study population consisted of adults aged ≥40 years residing in the United States with access to a smartphone and a Fitbit (Google) activity tracker. Recruiting was conducted through MHealthy and UMHealthResearch, 2 web-based platforms at our university focused on wellness and health research, respectively. Recruitment emails containing a detailed study description, contact information, and a link to the Qualtrics eligibility screener (Qualtrics International Inc) were sent to potential participants who expressed interest in the study.

The eligibility screening involved a conditional 2-item self-report measure assessing participants’ satisfaction with their current activity levels. The first question asked the following—*I want to be more physically active*—with responses on a 5-point Likert scale ranging from *strongly disagree* (1) to *strongly agree* (5). Participants who scored ≥3 were then shown the second question—*To be more active, I want to increase how much I walk*—also rated on the same 5-point scale. Participants who scored ≥3 on both items (ie, indicating low satisfaction with their current activity level and strong motivation to increase PA through walking) were invited to participate.

This proof-of-concept study intentionally included participants across a wide range of baseline activity levels rather than restricting recruitment to sedentary individuals to assess the feasibility of WalkToJoy broadly. In addition, due to feasibility constraints (ie, limited funding), we recruited individuals who already owned a Fitbit and expressed interest in becoming more active but struggled with maintaining enjoyment and consistency. Eligible participants were subsequently asked to confirm their intent to take part and complete an informed consent form. Upon completion, they provided their email address and phone number to receive further instructions for study enrollment. The inclusion criteria are summarized in Textbox 1.

**Inclusion criteria.**

- Adults aged ≥40 years living in the United States
- No medical condition that would prevent them from safely engaging in a moderate physical activity (ie, walking)
- Access to a smartphone that could run the Fitbit app
- Possession of and willingness to use a Fitbit daily for the duration of the study
- A safe and easily accessible place to walk
- Low satisfaction with current physical activity level and desire to increase activity by walking more

### Ethical Considerations

This study was approved by the Health Sciences and Behavioral Sciences Institutional Review Board at the University of Michigan (HUM00217566). All participants provided informed consent electronically via a secure Qualtrics survey, which included study details, contact information, and information about voluntary participation and withdrawal rights. Participants could withdraw at any time and request deletion of their data, which was honored immediately. To ensure confidentiality, each participant was assigned a unique ID, and all survey and Fitbit data were linked only to these IDs. Personally identifiable information (eg, names, emails, and phone numbers) was stored separately in secure, access-restricted databases. A subset of participants was invited to optional Zoom interviews, which were recorded, transcribed, deidentified, and securely stored. Participants were compensated up to US $52 based on their level of participation.

### Study Procedures

**Figure 3:**
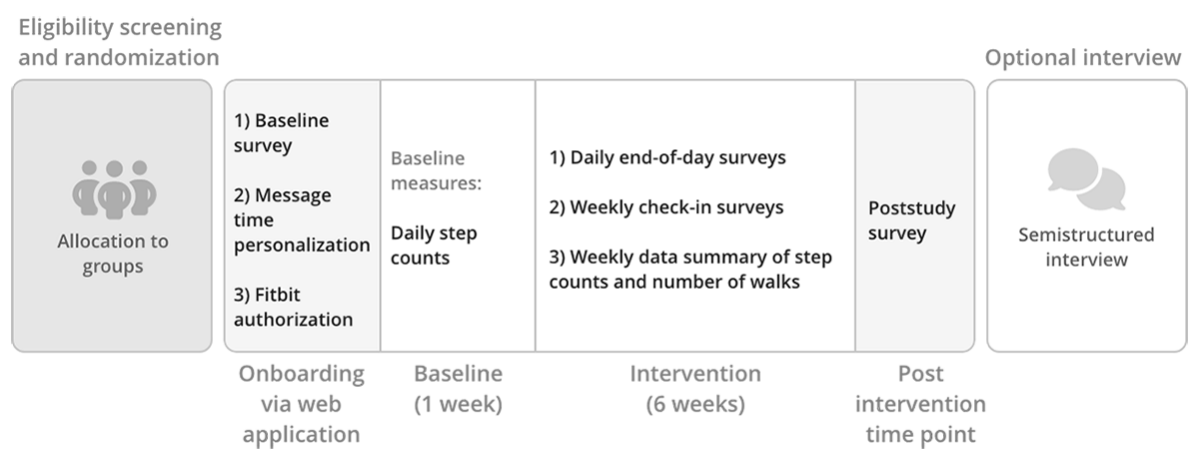
Study flow diagram.

Participation in the study involved several steps: onboarding through the WalkToJoy web application, a 1-week baseline assessment, a 6-week intervention period, a postintervention assessment, and an optional semistructured interview. The overall study flow is illustrated in Figure 3 and described in detail in the following sections.

Due to COVID-19 restrictions, the study was conducted entirely remotely. Upon providing consent, eligible participants were randomly assigned to 1 of 8 study conditions and received a participant ID, account token, and detailed instructions via email. Using these credentials, participants logged into the WalkToJoy web application, accessed through a mobile web browser on their smartphones, to complete the onboarding tasks and the baseline survey.

**Figure 4:**
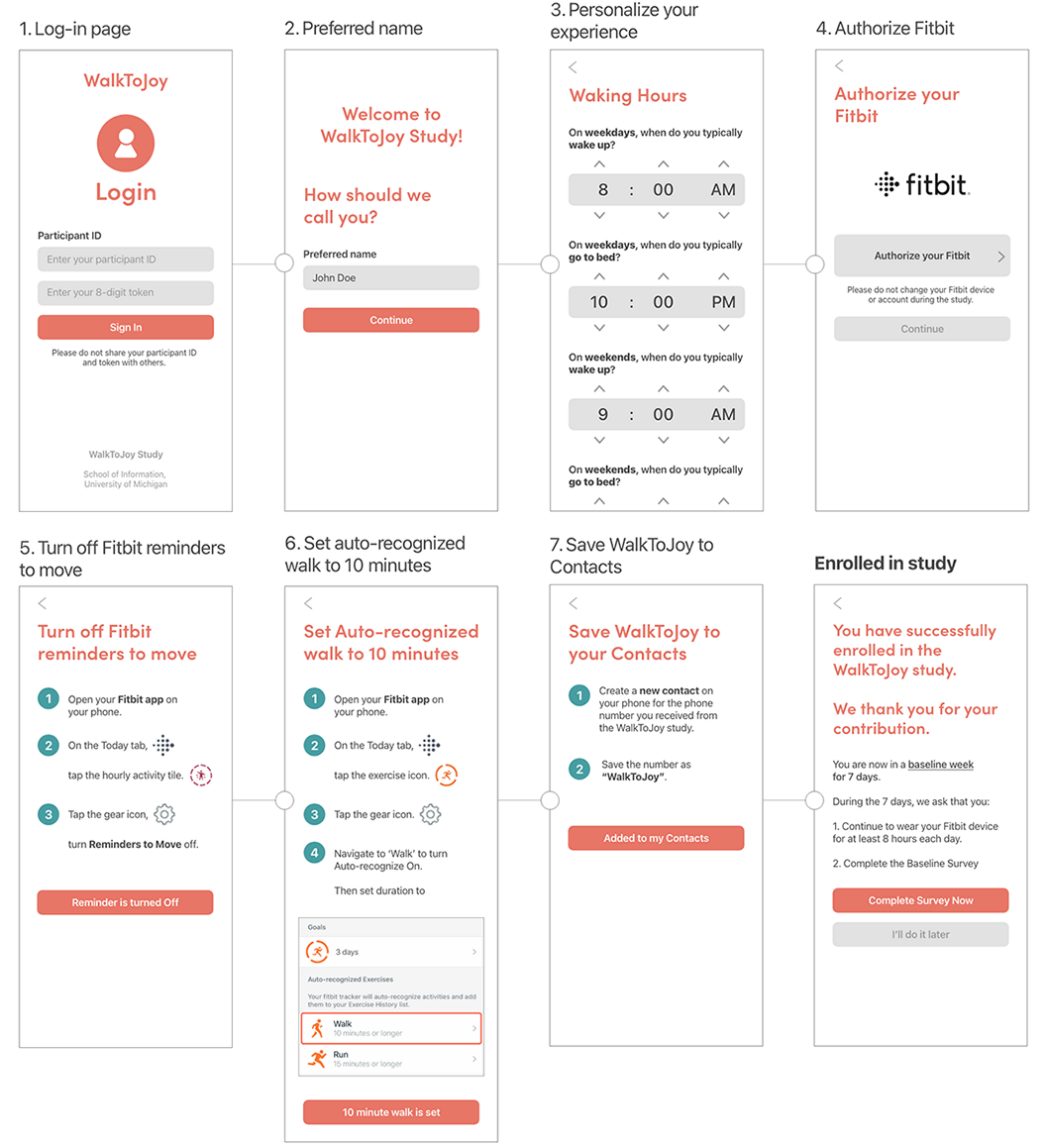
Onboarding application interaction map.

During onboarding, participants personalized their experience by entering their preferred name and typical waking and sleeping hours, connecting their Fitbit account, and configuring Fitbit and phone settings. Figure 4 provides an overview of this onboarding workflow.

As part of the baseline survey, participants provided demographic and psychosocial information, including gender; motivation for PA; and baseline measures of affective attitudes toward and affective valuations of walking, exercise self-efficacy, and body awareness. Fitbit data were collected for a minimum of 7 days before the start of the intervention to establish baseline step counts. Participants who wore their Fitbit for at least 3 days (≥8 hours per day) during the baseline week transitioned into the intervention phase the following Monday. Those who did not meet this wear threshold were sent reminders and given up to 7 additional days to meet the baseline criteria.

### Intervention Components

#### Overview

The study assessed 3 components as part of the WalkToJoy intervention package. All intervention messages were delivered via SMS text message or Multimedia Messaging Service, with delivery status and time stamps logged on the WalkToJoy server. No real-time message adaptation (eg, context-aware triggering or dynamic content) was implemented; all messages were prescheduled based on personalization and randomized across participants. Participant activity was monitored using minute-level Fitbit data accessed through the Fitbit application programming interface.

#### Positive Conditioning via Walking Suggestion GIF Prompts

**Figure 5:**
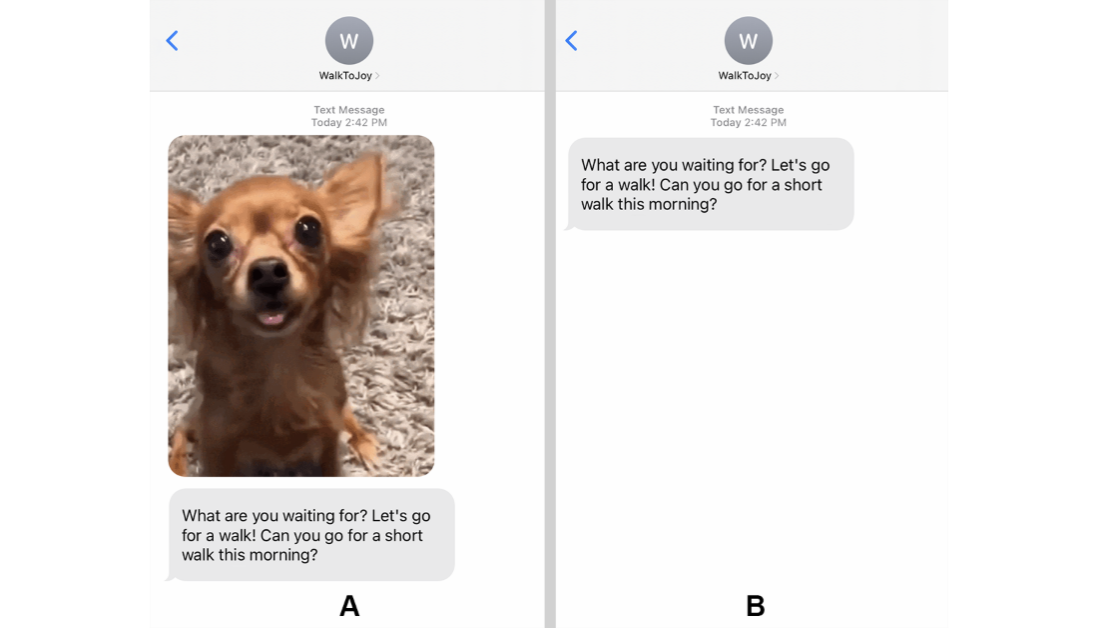
An example walking suggestion for the (A) GIF group and (B) non-GIF group.

The first intervention component involved sending participants smile-inducing messages suggesting they go for a walk within the following few hours (Figure 5). These SMS text messages were either accompanied by an animated GIF image designed to elicit automatic positive affect (GIF group) or sent without a GIF image (non-GIF group).

#### Salience Messages About Positive Aspects of Walking

**Figure 6:**
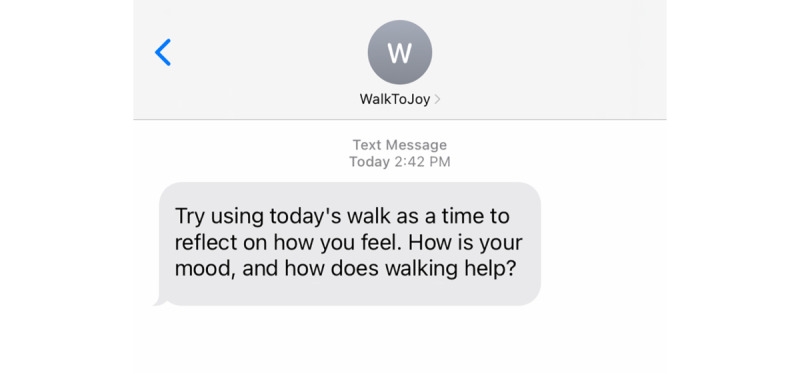
An example salience message.

The second intervention component involved sending salience messages (Figure 6) that directed participants’ attention to the positive aspects of walking. The message content was randomly selected from topics such as observing the environment, focusing on the body, noticing affective states, or engaging in an enjoyable activity while walking.

#### Planning Prompts to Reflect on and Plan Enjoyable Walks

The third intervention component involved sending a weekly reflection prompt on Monday mornings asking participants to reflect on the previous week’s walking experience and create an actionable plan to make future walks more enjoyable. The prompt provided examples of strategies such as listening to a favorite album or podcast, walking with a pet or family member, or trying a new route. Participants were randomly prompted to either try a new planning strategy or continue the one from the previous week. After completing the reflection, participants received reminders twice a week (Tuesdays and Thursdays) to follow through with their plans.

### Randomization

The WalkToJoy intervention package was evaluated for feasibility and adherence using a pretest-posttest study design. To maximize what we could learn from this study and assess the preliminary efficacy of each intervention component in improving participants’ affective valuations and attitudes toward walking over the 6-week study, a 3 × 2 factorial design was implemented. For walking suggestions, the factor levels were receiving walking suggestions *with* cute animal GIF images (factor *on*) or receiving walking suggestions *without* the GIF images (factor *off*). For salience messages, the factor levels were receiving the salience messages (*on*) or *not* receiving the salience messages (*off*). For planning, the factor levels were receiving the planning prompts (*on*) or *not* receiving the planning prompts (*off*). Upon enrollment, participants were randomized across the 3 factors, resulting in assignment to 1 of 8 unique intervention conditions (Table 1).

**Table 1:** Intervention conditions.

Condition	Walking suggestion	Salience message	Planning prompt
1	On^a^	On^a^	On
2	On^a^	Off	On
3	On^a^	On^a^	Off
4	On^a^	Off	Off
5	Off^a^	Off	Off
6	Off^a^	On^a^	On
7	Off^a^	On^a^	Off
8	Off^a^	Off	On

^a^Embedded microrandomized trials.

In addition, the study included microrandomization of the walking suggestions and salience messages to evaluate their near-term impact on step counts within a 4-hour window and on daily affective reflection about their recent walks, respectively.

The WalkToJoy system microrandomized the delivery of GIF images and text-only walking suggestions at 2 decision points per day (ie, morning and afternoon) determined by participants’ wake-up times and time zones, with a 50% probability of activation at each decision point. This meant that participants were expected to receive, on average, 1 walking suggestion per day. Randomization at each decision point was independent of intervention provision at previous decision points. Given the study duration of 42 days, each participant could be randomized up to 84 times.

For participants in the salience conditions, salience messages were microrandomized with a 50% probability once per day at noon based on participants’ time zone. As a result, participants would receive, on average, a salience message every 2 days. Participants in the nonsalience conditions did not receive any salience messages.

There was no embedded MRT for the planning component. Participants in the planning conditions received a planning prompt each Monday morning once a week with an activation chance of 100% but randomly received 1 of the 2 types of planning prompts with 50% probability: a prompt to try a new planning strategy or a prompt to keep the same strategy as the previous week. After completing the planning prompt, participants received two reminders during the week (on Tuesdays and Thursdays) reiterating the specific plans they had written. Participants in the nonplanning group did not receive any planning prompts or reminder messages.

### Outcome Measures

#### Primary Outcome Measure: Affective Attitudes

Table 2 presents a summary of the outcome measures and assessment times. The primary outcome measure consists of a modified 7-point, semantic differential 3-item measure of affective attitude toward walking [57-60] administered through the weekly check-in survey. The specific items are shown in Textbox 2.

**Table 2:** Summary of the outcome measures and assessment times.

Outcome measure	Baseline phase	Intervention phase (wk 1-6)	Post study time point
Affective attitude	✓	✓^a^	✓
Affective valuations	✓	✓^a^	✓
Affective reflection		✓^b^	
Anticipated affect		✓^b^	
Step counts	✓	✓^c^	
Exercise self-efficacy	✓		✓
Body awareness	✓		✓
Poststudy intervention evaluation and barriers			✓

^a^Collected weekly.

^b^Collected daily and weekly.

^c^Collected daily and at the minute level.

**Affective attitude scale.**

- *For me, walking is...*: response options from *very unpleasant* (1) to *very pleasant* (7)
- *For me, walking is...*: response options from *unenjoyable* (1) to *enjoyable* (7)
- *For me, walking is...*: response options from *boring* (1) to *interesting* (7)

By measuring affective attitude on a weekly basis, the items capture the changes in participants’ affective experience of walking over the course of the study. The measure was selected as the primary outcome because (1) it would be impacted if the intervention were effective and (2) it is a key mechanism of change postulated by ART.

#### Secondary Outcome Measure: Affective Valuations

The secondary outcome measure consists of a modified 7-point, semantic differential 1-item measure of affective valuations [57,60] administered via the weekly check-in survey. The item was phrased as follows—*When I think about walking...*—with response options anchored between *I cringe* (1) and *I smile* (7).

#### Exploratory Outcome Measures

In addition to the primary and secondary outcome measures, the study assessed daily step counts via Fitbit-measured steps, as well as affective reflection and anticipated affect on a daily and weekly basis to assess daily and weekly changes in participants’ views on previous and upcoming walks. The weekly measure of affective reflection consisted of a 7-point, semantic differential 1-item instrument that asked the following—*How did you feel overall when walking this last week?*—with answers anchored between *I couldn’t wait for it to end* (1) and *Loved every second of it!* (7). The daily affective reflection measure consisted of a 7-point, semantic differential 1-item instrument that asked the following—*How did you feel overall when walking today?*—with answers anchored between *I couldn’t wait for it to end* (1) and *Loved every second of it!* (7). The weekly anticipated affect [58,59] measure consisted of a modified 7-point, semantic differential 1-item instrument that asked the following—*This coming week, I expect walking will feel...*—with answers anchored between *Awful!* (1) and *Great!* (7). Finally the daily anticipated affect measure consisted of a modified 7-point, semantic differential 1-item instrument that asked the following—*Next time you take a walk for 5 minutes or longer, what will that be like for you?*—with answers anchored between *Awful!* (1) and *Great!* (7).

#### Proximal Outcome Measures for Microrandomized Factors

The proximal outcome measures for the MRT of walking suggestions for both the GIF and non-GIF groups was the number of steps taken within 4 hours following each randomization. Our expectation was that participants would take more steps within 4 hours after receiving a walking prompt compared to when they did not receive one and that GIF prompts would be more effective than non-GIF prompts. The proximal outcome for salience messages was participants’ affective reflections about their walks that day and anticipated affect for upcoming walks, measured at the end of each day.

#### Additional Measures of Potential Effect Modifiers

The Exercise Self-Efficacy scale was administered before and after the intervention to evaluate participants’ self-efficacy toward exercise behavior as a potential moderator of study outcomes. The Exercise Self-Efficacy Scale [61] includes 9 self-report items measured on a 4-point response scale from *Not at all sure* (1) to *Very sure* (4).

In addition, a reduced version of the Revised Body Awareness Rating Questionnaire was administered before and after the intervention to evaluate participants’ awareness of their own bodies. The reduced version of the Revised Body Awareness Rating Questionnaire [62,63] includes 7 self-reported items measured on a 4-point response scale from *Completely disagree* (0) to *Completely agree* (3).

#### Poststudy Measures

At the end of the intervention, participants were prompted to complete a poststudy survey to evaluate their experiences with the intervention and barriers to participation. The measures consisted of questionnaires covering the following topics: (1) general attitude toward the intervention, (2) participants’ subjective evaluation of the intervention (ie, whether it was beneficial and improved affective attitude toward walking), (3) participants’ subjective evaluation of the intervention’s usability (ie, whether it was easy to incorporate into their daily lives, there were too many messages, and the guidelines were clear), and (4) any barriers to participating in walks and surveys.

#### Adherence Protocol

All participants received a minimum of US $10 to a maximum of US $52 via a check for participating in the study based on their adherence to study assessments and Fitbit wear time. To ensure the reliability of study data and participant engagement throughout the intervention, adherence protocols were implemented for the baseline survey, daily and weekly outcome surveys, and activity data monitoring.

For the baseline survey, participants received reminder messages 24 and 48 hours after enrolling if the survey was not completed. Participants were dropped from the study if they failed to respond within the 7-day baseline period. Weekly surveys were sent every Sunday at 8 PM, with up to 2 daily reminders for noncompletion. Daily end-of-day surveys were sent at 8 PM (excluding Sundays), with reminders sent after 2 consecutive missed days. If surveys remained incomplete, a phone call from a study coordinator was made on the fifth day. Activity adherence was monitored by tracking Fitbit use. Participants received up to 3 reminders starting with 2 consecutive nonwear days. After 6 consecutive nonwear days, a study coordinator contacted the participant via phone.

#### Semistructured Interviews

The multifaceted design of the intervention study, incorporating both factorial and microrandomized approaches, enabled the assessment of participants’ varied experiences with different combinations of intervention components. These components differed in the frequency of notifications, prompts, and tasks delivered to participants’ phones. At the end of the study, one participant from each intervention group was randomly selected to participate in an optional semistructured interview that lasted 45 to 60 minutes to gather qualitative data on their experiences with incorporating walking into daily life, perceptions of the value of the intervention components, and barriers to participation (see Multimedia Appendix 1 for the interview protocol). Using purposive sampling, participants were selected based on their allocation to 1 of the 8 intervention arms and their recent completion (within 30 days) of the 6-week study. If a selected participant declined, another was randomly chosen as a replacement. These interviews were conducted individually and audio recorded through the videoconference software Zoom (Zoom Video Communications). The recordings were transcribed and deidentified for further analysis.

### Statistical Power

In line with the Obesity Related Behavioral Intervention Trials model for behavioral treatment development [64], this study was designed as a proof-of-concept trial with the goal of evaluating whether the WalkToJoy intervention package showed promise that it could meaningfully impact a key affective mechanism—affective attitudes—that mediate sustainment of PA. Therefore, our aim was to explore whether the intervention showed signs of potential efficacy to guide future refinement and scaling efforts. This approach aligns with the Multiphase Optimization Strategy framework, which encourages early-phase experimentation to assess feasibility and identify promising intervention components to inform package optimization before larger trials, such as randomized controlled trials [54,65]. At the time of planning the study, there was no clear empirical evidence for what magnitude of change in affective attitudes should be considered clinically meaningful. As a result, we relied on an observation that a change of half an SD constitutes a meaningful impact across a range of health measures [66,67]. Starting with the SD for affective attitudes of 1.21 reported by Kiviniemi et al [60], we calculated that an impact of half an SD would correspond to a moderate standardized effect size of 0.5. To detect this effect size with 90% power and type I error rate of .05, we calculated that we needed a minimum of 36 participants. A recently published paper by Dunton et al [68] has since provided further support for these assumptions by finding that even a change of a third of an SD in affective response to exercise can meaningfully improve exercise behavior. Assuming a 10% attrition, as in our recent mHealth studies, the minimum required sample size was 40 participants. We note that this sample size would have also enabled us to detect a small standardized effect of 0.12 on the proximal outcome of the microrandomized walking suggestions and a standardized effect of 0.17 on the proximal outcome of the microrandomized salience messages, both with 90% power at the α level of .05.

In addition, while step count was included as an exploratory behavioral outcome, this study was not powered to detect statistically significant between-group differences at the conventional α level of .05. Rather, the aim was to identify potential signs of effect that could inform future hypothesis-driven trials, consistent with the exploratory goals of early-phase behavioral intervention research.

### Statistical Analyses

To examine changes in affective attitudes (primary outcome), affective valuations (secondary outcome), and step counts (exploratory outcome) between the start and end of the study, we used descriptive statistics and paired 2-tailed *t* tests. In addition, to assess the main effects of each of the 3 baseline-randomized factors on the weekly assessments of the affective measures as well as daily measures of step count, we used the generalized estimating equations (GEE) model. This allowed us to assess the overall (average and across time) effect of delivering the GIF version of the walking suggestions versus delivering the non-GIF, text-only version of the suggestions; the overall effect of delivering salience messages about positive aspects of walking versus not delivering salience messages; and the overall effect of prompting weekly planning of enjoyable walks versus not prompting planning. To decrease noise, covariates in our analyses included time in the study and baseline values of the outcome variables (affective measures and step counts, respectively). For the GEE analyses of the factorial experiment, 1-sided *P* values were used to test directional hypotheses that the presence of each intervention component (GIF prompts, salience messages, and planning prompts) would lead to improvements in affective outcomes and step count. This decision reflects the study’s proof-of-concept nature, where the aim was to maximize sensitivity to detect expected beneficial effects rather than assess bidirectional changes.

The analyses of microrandomized components examined their impact on their respective proximal outcomes. For walking suggestions, our primary interest was the average effect of sending a prompt versus not sending a prompt on the subsequent 4-hour step count. The 4-hour window of the proximal outcome was anchored in the time of message randomization. For the salience messages, our primary interest was the average effect across time of sending a salience message versus not sending a salience message on the affective reflection (ie, enjoyment) on the day’s walking experience and anticipated affect toward upcoming walks measured at 8 PM each evening. To assess these effects, we used the centered and weighted least squares method [12,69], the standard statistical approach for examining data from MRTs. This approach enables robust estimation of causal treatment effects while accommodating longitudinal data correlations through the nesting of outcomes within participants [69]. To reduce noise, the analyses of the walking suggestions included a covariate of the 1-hour prerandomization step count.

In addition to the statistical analyses, the data from the postintervention interviews were thematically analyzed using an inductive approach, beginning with a detailed review of each transcript to identify patterns and assign unique codes. Emergent themes and subthemes were synthesized through systematic rereading and joint discussions among the research team to ensure rigor and resolve discrepancies. The analysis concluded when data saturation was reached, indicated by the absence of new themes or variations.

## Results

### Participant Sample

**Figure 7:**
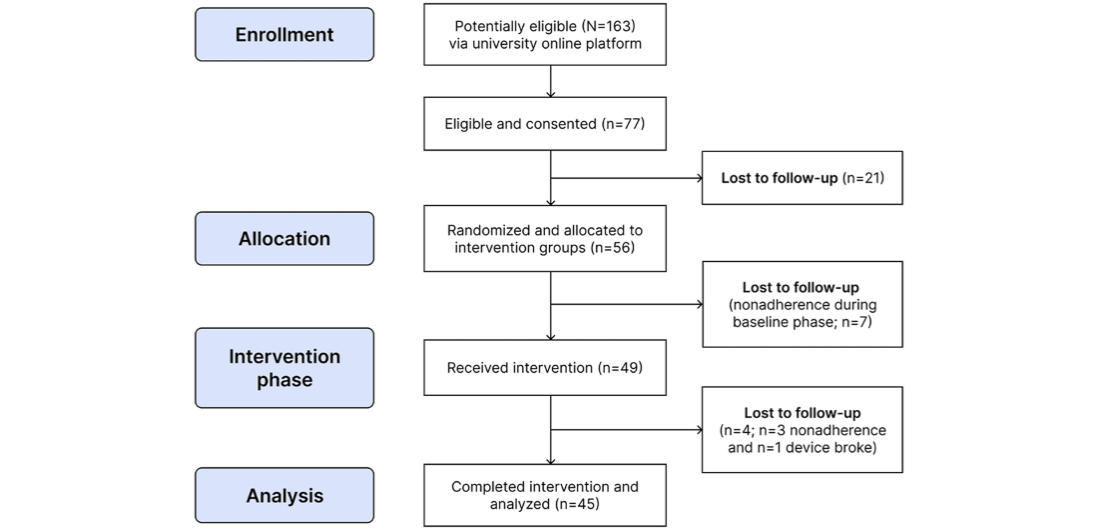
CONSORT (Consolidated Standards of Reporting Trials) flow diagram.

Participant recruitment began in May 2023, with 77 individuals initially consented via the university online platform. However, of these 77 individuals, 21 (27%) were lost to follow-up during onboarding to the WalkToJoy app and never progressed to the baseline phase. In total, 56 participants were onboarded and randomized to the intervention groups, but 7 (12%) were lost to nonadherence (ie, they did not wear the Fitbit activity tracker during the baseline phase). Ultimately, 49 participants received the intervention, but 4 (8%) more were withdrawn due to nonadherence to wearing the Fitbit activity tracker (ie, 10 consecutive days of no activity data despite reminder messages and emails; n=3, 75%) or because their device broke (n=1, 25%). A total of 45 participants successfully completed the study (Figure 7). Data collection concluded in September 2023. The demographic characteristics of the 49 participants who received the intervention are summarized in Table 3.

**Table 3:** Demographic characteristics of the WalkToJoy participants (N=49).

Variable (categorical)		Participants, n (%)
**Gender**		
	Woman	40 (82)
	Man	9 (18)
**Age range (y)**		
	40-49	24 (49)
	50-59	12 (24)
	60-69	8 (16)
	≥70	5 (10)

The recruitment for exit interviews involved a purposive sampling strategy to select participants who (1) were allocated to 1 of the 8 intervention arms and (2) had recently (within the previous 30 days) finished taking part in the study. Of the 14 participants invited, 9 (64%) completed the interview process. Notably, an additional participant was invited from the subgroup that received the comprehensive intervention package comprising all 3 components. The demographic details of the interview participants are summarized in Multimedia Appendix 2.

### Adherence and Data Missingness

Of the 45 participants who completed the study, 40 (89%) completed the affective measures for the primary and secondary outcomes (affective attitude and affective valuations) at both baseline and the final intervention week; pretest-posttest statistics are reported for this subset. In addition, 93% (42/45) of the participants provided valid Fitbit wear data—defined as at least 3 days per week with ≥8 hours per day of wear, confirmed via heart rate minute data—at both baseline and the final intervention week (week 6). Pretest-posttest analysis for average daily step count is reported for this subset.

Among the 45 participants enrolled in the study, adherence to the weekly outcome assessments varied across the 6-week intervention period. A total of 270 weekly assessments were expected across all participants (45 participants × 6 weeks = 270 total assessments). Overall, 94.1% (254/270) of the expected assessments were completed, indicating a high level of adherence. The percentage of participants who completed all 6 assessments was 76% (34/45), whereas 98% (44/45) completed at least 4 out of the 6 assessments.

The total possible decision points for the microrandomized GIF and text-only walking suggestions were 3240 (45 participants × 36 days × 2 per day), where participants received suggestions 2 times per day excluding Sundays. Of these 3240 possible decision points, 25 (0.77%) were not randomized due to technical issues, resulting in a total of 3215 (99.23%) decision points randomized. Across the 3215 decision points, both heart rate data and step count data for the proximal outcome were completely missing in 217 (6.75%). This missingness likely resulted from device nonwear during the entire proximal outcome time frame (ie, a 4-hour window). Among the remaining 2998 decision points, heart rate data were partially missing in 335 (11.17%), indicating intermittent wear or data transmission issues during that window. To address wear adherence missingness, an imputation method was applied for the MRT analyses of the walking suggestions. For each minute within the 4-hour proximal outcome window following a decision point, heart rate data were assessed within a 5-minute radius. If data were detected within this window, the minute was classified as an *adherent minute* even if no heart rate data were available for the exact minute.

The total possible number of decision points for the microrandomized salience messages was 792 (22 participants × 36 days × 1 per day), where the 22 participants randomized to the salience group received salience messages once per day excluding Sundays. Of these 792 possible decision points, 46 (5.8%) were not randomized due to technical issues, resulting in a total of 746 (94.2%) decision points randomized. Of the 746 decision points, 381 (51.1%) were randomized to send salience messages, and 365 (48.9%) were randomized to not send any salience messages that day. Of the 381 activated salience messages, 1 (0.3%) failed to be delivered due to mobile carrier network issues. In addition, across these 745 decision points, missingness occurred in 103 (13.8%) for the measure of affective reflection and anticipated affect when participants failed to complete the daily end-of-day survey for that day. Therefore, the MRT analysis of the salience factor was conducted on the 642 decision points with a valid proximal outcome.

### Changes Over Time for All Participants

#### Pretest-Posttest Differences in Affective Measures and Step Count

The average for affective attitude (primary outcome) significantly increased by 0.547 points on a 7-point scale from baseline to the final week of the study (*P*<.001), with a gradual trend on a weekly basis (Table 4). The average for affective valuations (secondary outcome) showed an even greater improvement, increasing by 0.718 points on a 7-point scale (*P*<.001). In addition, the exploratory outcome measures of affective reflection and anticipated affect both showed a significant increase of 0.692 points (*P*<.001 for both).

**Table 4:** WalkToJoy pretest-posttest measurements.

	*t* test (*df*)	*P* value	Mean difference (95% CI)
Affective attitude	4.139 (38)	<.001	0.547 (0.279-0.815)
Affective valuations	5.233 (38)	<.001	0.718 (0.440-0.996)
Affective reflection	4.004 (38)	<.001	0.692 (0.342-1.042)
Anticipated affect	4.423 (38)	<.001	0.692 (0.375-1.009)

In addition to affective measures, we explored changes in valid average daily step count (exploratory outcome). The primary pretest-posttest analysis comparing baseline to the final intervention week (week 6) showed a nonsignificant increase of 80 steps (*P*=.79). However, visual inspection of the weekly step data revealed a peak in week 5 followed by a drop in week 6, which appeared to be influenced by a small number of extreme outliers. As a post hoc exploratory analysis, we calculated the change from baseline to the average of weeks 4 to 6, which showed a marginal 506-step increase in average daily steps (*P*=.07).

#### Participant Perceptions of the Impact of the Intervention on Affective Associations With Walking

Thematic analysis of exit interviews suggested that the intervention successfully impacted participants’ affective associations and engagement with walking. Across varying intervention exposures, participants consistently reported heightened awareness of the positive aspects of walking, fostering positivity, confidence, and motivation. A participant explained the following:

I got to be more aware...I was kind of looking forward to the opportunity. Okay, I’m out of my desk. I’m gone. Let’s go...I was just feeling content and comfortable with it.

P24

Participants reported that this increased awareness and intention led to an integration of walking into their daily routines. One participant described the following:

...it’s been a couple of weeks since I stopped the study...and I still feel that routine. I’m more amenable to doing actual walks for exercise.

P7

As walking became more of a routine, participants’ attitudes shifted, transforming walking from a task to an anticipated and enjoyable experience. Psychological barriers diminished over time, making walks feel more accessible. One participant illustrated this shift:

...as I progressed...I started looking forward to it. And then as I’m walking, I’m like, la-di-da.

P24

Overall, participants reported that the intervention fostered cognitive and emotional alignment with the benefits of walking, reinforcing habit formation, increasing enjoyment, and strengthening participants’ intention to walk regularly despite potential obstacles.

### Effects of Individual Intervention Components

As walking prompts, salience messages, and planning were baseline randomized in a factorial experiment, we examined the effects of each of the 3 intervention components on both affective measures and steps. A GEE model was used to account for within-subject correlations over the 6-week intervention period.

#### GIF Versus Text-Only Walking Suggestions

From baseline to the end of study, the delivery of smile-inducing GIF walking suggestions resulted in a nonsignificant increase of 0.099 points in affective attitudes (*P*=.37) and of 0.117 points in affective valuations (*P=*.33) compared to text-only prompts. The anticipated affect toward future walks (an exploratory outcome) showed a statistically significant increase of 0.345 points (*P=*.046) from baseline to the end of the study for the GIF group compared to the text-only prompt group. These findings suggest that GIF prompts were a key driver of the positive shift in participants’ anticipation of walking, potentially mediated by the evaluative conditioning effects of smile-inducing stimuli. In addition, GIF walking suggestions significantly influenced PA. Participants in the GIF group walked, on average, 1834 more steps per day over the course of the study than participants in the text-only group (SE 1092; P=.05), based on a GEE model adjusting for within-subject correlation.

#### Salience Messages and Planning Prompts

No meaningful increases were observed in affective measures or step counts when comparing the salience group to the nonsalience group or the planning group to the nonplanning group. This may indicate the need for further refinement of these components, longer intervention periods, or larger sample sizes to better assess their independent effects.

#### Participant Perceptions of GIF Walking Prompts as Drivers of Engagement

In alignment with the factorial analysis, the thematic analysis of participant interviews revealed a notable difference in the perception and reception of smile-inducing GIF versus text-only walking suggestions. Participants in the non-GIF group acknowledged the text-only walking prompts as helpful reminders but reported message fatigue over time, particularly when compounded with multiple reminders and other notifications. One participant noted the following:

The daily reminders and encouragement weren’t really encouraging to me and I’ll tell you why...it just becomes old news.

P24

Similarly, another participant explained the following:

They were just good gentle reminders that, you know, let’s go for a walk today. [...] I think what I found a little bit annoying is when I was getting multiple reminders in a day. I mean, I don’t want to turn off the texts completely. I still wanted to get texts, but I think sometimes it just felt like too many in a day.

P33

In contrast, participants in the GIF group consistently described the prompts as enjoyable, highlighting their humor and visual appeal. One participant shared the following:

It made me happy and brought my mood up and it made everything a lot easier to do because it was funny.

P32

Another participant echoed this sentiment:

I have to say the texts helped a whole lot. It definitely wasn’t stressful to receive the prompt, and it helped that it had something visually appealing to look at, whether it was funny or motivating, you know, you can’t really get upset at a text like that...it’s entertaining for the 30 seconds and I’m like, okay, I gotta walk.

P5

In addition, the smile-inducing GIF prompts appeared to foster a sense of positive anticipation that sustained engagement throughout the study. One participant reflected the following:

Yes, the little dogs [chuckles]...it’s just that little nudge that you need sometimes when you’re like, I don’t really feel like it, but I know I should. So, getting the message made it nice. [...] it was just a fun way to get going in the morning.

P6

This sense of expectancy contributed to the intervention’s ability to sustain motivation and engagement even on days when prompts were not delivered due to the microrandomization process. Several participants described an evolving relationship with the prompts, initially dismissing their relevance but later expressing appreciation for their impact. One participant recounted the following:

Like initially I was kind of like, oh, how silly...it’s like this little animal or whatever. [...] But I have to admit [chuckles], by the end of the study, I was always kind of curious to see the next one...There was one that was just great, it was like this dog that was jumping on all fours, up and down, ready to go for a walk, right [chuckles]. I liked it so much.

P7

Participants also indicated that the GIFs prompted mindfulness regarding walking and subtly integrated it into their daily routines. One participant explained the following:

I’ve internalized your memes and your messages. I do try to get more walks in. I know that I’m a little bit more conscientious about the time. So around 11 o’clock, I’ll look at my phone, or I’ll look at where I’m at, and be like, you know, this is a good time to take a break. You know, and I’ll go out and do something.

P5

#### Participant Perceptions of Salience Messages as Catalysts for Attitudinal Change

Despite the non–statistically significant quantitative results, the thematic analysis revealed that salience messages may have served as a catalyst for attitudinal change by shifting participants’ perceptions and experience of walking from obligation to opportunity. In the group that received salience messages (ie, the salience group), participants reported a positive shift in attitude toward and experience of walking. Several participants distinctly recalled specific messages that impacted them. For instance, one participant noted the following:

I like the ones especially about, the one that has stuck with me the most, is the one that you just said about giving you more energy. Like, are you tired? Go for a walk. It will give you more energy.

P33

When asked about their change in experience of walking as a result of reading the salience messages, one participant explained the following:

...we were truly walking around and like looking at different things and enjoying the outdoors...like I tried to be more aware of how it impacted me.

P3

Another participant echoed this sentiment:

One of the messages, you know, kind of suggested, slowing down, you know, and it’s so counterintuitive. That one, it was a very positive message, and it, for me, personally, it worked. I was approaching walking a little bit weird, it was always like this mad dash rush, a thing to get it done and whatever, but...ever since I read the message, it sort of resonated with me...And I remember, just a change in how I was approaching the whole thing a little bit and taking my time with it. It’s something I’ll probably keep going forward with having that in mind.

P7

For P7, the salience messages reframed the expectation of walking from a chore to an opportunity for stress relief and a step away from the hustle of daily life by fostering a sense of mindfulness and introspection about their walking experiences. Similarly, another participant noted an increased awareness of the physical and mental benefits experienced after walking, suggesting a heightened appreciation for its value:

It really made me pay attention to my body and how it functions. [...] I think a lot of times in life we forget about, you know, the important things in life and the fresh air and the flowers and all that stuff really helps, you know. And it increases your mood as well, you know.

P32

#### Participant Perceptions of Planning Prompts as a Way to Foster Enjoyable Walking Experiences

Despite the non–statistically significant quantitative results, the interview findings revealed that participants who received planning prompts and reminders found them helpful to reflect on ways to make walking more enjoyable. As noted by one participant, the process of planning allowed participants to personally tailor their walking experiences, fostering an interactive and pleasurable engagement with walking:

I really like to listen to music when I walk, so I made sure that I have my headphones charged and I had my, you know, my phone charged up, so I could just be kind of by myself listening to my music. That was nice, that was really nice.

P6

This not only enhanced the enjoyment of their walks but also boosted their motivation and overall mood after the walks, as noted by one participant:

I tried the different ones and I think the best one would be, well, I tried to listen to an album and that help with the motivation and make my steps a little bit faster...And I also enjoyed one, you know, just to be mindful and have a lot of awareness of my environment. And those definitely increased my mood and motivation.

P32

This selection process was complemented by customized reminder messages, which kept their weekly chosen strategies on top of participants’ minds, thereby supporting sustained engagement and motivation throughout the study. One participant explained the following:

...what was motivating for me is when it was personalized. And even at the end of the week, even though it was simple, “you said last week you were going to do this.” It engaged me more.

P42

In addition, P42 noted how they desired an even more personalized interaction based on their chosen strategy for each week. They added the following:

But, if you’re able to customize it to, “what plant did you see today?” You know, on Wednesday, send me a message, but not overdo it. Find a balance.

P42

### Effects of Microrandomized Intervention Components

The MRT analyses of walking suggestions did not show a significant difference in step counts in the 4-hour window following the decision points, either between providing any kind of walking prompt and providing no prompt or between sending a GIF prompt versus a text-only walking prompt (*P*=.55). For the salience group, the MRT analysis revealed a nonsignificant increase in daily affective reflection scores of 0.142 points when salience messages were sent compared to when no message was sent (*P*=.26). Similarly, there was a moderate but nonsignificant increase in daily anticipated affect toward upcoming walks of 0.16 points (*P*=.15).

## Discussion

### Principal Findings

The primary aim of this proof-of-concept study was to evaluate the feasibility of the WalkToJoy intervention and gather initial evidence for its ability to have clinically meaningful impact on affective association with PA and on PA levels among middle-aged to older adults. The intervention package was grounded in the ART of physical inactivity and exercise, and it leveraged both automatic and reflective affective processes to foster intrinsic motivation [20,28]. Our secondary aim was to gather preliminary evidence for the efficacy of each of WalkToJoy’s 3 intervention components—smile-inducing walking suggestions, salience messages, and planning prompts—to inform optimization decisions for their use in future versions of the WalkToJoy intervention.

Table 5 combines both the quantitative and qualitative findings. Overall, the results suggest that WalkToJoy is both feasible and promising for fostering positive affective associations with walking and promoting engagement with the walking behavior itself. The intervention package as a whole demonstrated robust pretest-posttest improvements in affective outcomes. Affective attitude, the primary outcome, significantly increased by 0.547 points (*P*<.001), whereas affective valuations, the secondary outcome, showed an even greater improvement of 0.718 points (*P*<.001). Both of these impacts are over half an SD in magnitude, suggesting—based on recent findings [68]—that they may have a meaningful impact on engagement in PA itself. Exploratory measures, including affective reflection and anticipated affect, also demonstrated substantial gains, both increasing by 0.692 points (*P*<.001 in both cases). These findings underscore the intervention’s potential for impacting participants’ affective associations with walking, consistent with ART’s claim that repeated positive affective experiences can increase intrinsic motivation for PA.

Behaviorally, the pretest-posttest analysis of valid average daily steps showed a nonsignificant increase of 80 steps (*P*=.79). However, due to the presence of extreme outliers and a noticeable peak in step count during week 5, we conducted a post hoc exploratory analysis comparing baseline to the average of weeks 4 to 6. This revealed a larger increase of 506 steps (*P*=.07). While this finding suggests a preliminary signal of a positive behavioral trend, it should be interpreted with caution given its post hoc nature and the lack of significance at the conventional threshold for statistical significance. Nonetheless, the observed trend suggests a potentially meaningful signal worth exploring further in future optimization trials. An increase of 500 steps per day has been associated with a 6% reduction in cardiovascular disease risk and a 7% reduction in cardiovascular mortality [70,71], suggesting that this magnitude of change may have a meaningful implication for health promotion, particularly in midlife and older adults. Qualitative data further supported the positive impact on affective mechanisms and PA as well. Participants described heightened awareness of the benefits of walking; increased positivity, confidence, and motivation; and a diminished perception of psychological barriers.

Among the intervention components, the GIF walking suggestions emerged as the most impactful driver of affective and behavioral outcomes. While GEE revealed nonsignificant results for affective attitude and affective valuations, GIF prompts significantly increased anticipated affect toward future walks by 0.345 points (*P*=.046) compared to identical text-only prompts. These findings provide initial support for our hypothesis that evaluative conditioning—pairing a behavioral stimulus (eg, a walking prompt) with a positively valenced stimulus (eg, smile-inducing GIF images)—fosters positive emotional associations over time through automatic affective processing [28,37,72]. This conditioning mechanism likely contributed to the observed increases in participants’ positive anticipation and motivation to walk. In addition, GIF prompts significantly boosted steps, with GIF group participants averaging 1834 more steps per day across the entire study (*P*=.05) compared to those in the text-only group. This substantial behavioral impact highlights GIF messages’ potential as engaging and burden-resistant reminders that can impact PA behavior itself.

These results align with the qualitative insights, where participants showed a clear preference for the smile-inducing GIF walking prompts over identical text-only prompts for maintaining engagement and motivation. While text-only prompts were initially effective as reminders to walk, they became burdensome over time. In contrast, participants consistently described the GIF images as humorous, visually engaging, emotionally uplifting, and even evoking a sense of anticipation. These qualities helped sustain engagement and mitigate message fatigue. A recurring challenge in current mHealth interventions is achieving sustained user engagement, which is often corroded by treatment fatigue [14,73,74]. High user burden from repeated treatment delivery frequently results in low intervention adherence, thereby limiting the long-term effectiveness of such interventions. Our findings suggest that evaluative conditioning provides a promising framework for designing fatigue-resistant and positively valenced message delivery strategies. This approach has the potential to mitigate message fatigue and sustain engagement, especially in interventions requiring long-term adherence. Moreover, this affect-based approach could be extended to other components of mHealth interventions, such as planning, goal setting, and monitoring behaviors. Future studies should explore ways to integrate affective strategies into interventions that have traditionally relied on reflective cognitive processes to further enhance intervention effectiveness.

**Table 5:** Joint display of findings by each component.

	Quantitative findings	Qualitative insights	Integrated summary
All components	Pretest-posttest: significant increase on all 4 affective constructs (*P*<.001 in each case); increase of 506 average daily steps (*P*=.07) from baseline to the average of wk 4-6	Participants consistently reported heightened awareness of the positive aspects of walking, fostering positivity, confidence, motivation, and anticipation.	Findings demonstrate that the intervention strengthened affective associations with walking and may have produced a cumulative effect on behavioral outcomes.
GIF walking suggestion prompts	Factorial: anticipated affect increased by 0.345 points (*P=*.046), no significant effect on the other 3 affective constructs, and increase of 1834 average daily steps (*P*=.05); MRT^a^: no significant proximal effect on 4-h step count	Participants showed a clear preference for the GIF prompts over identical text-only messages, describing them as humorous, emotionally engaging, and enjoyable.	GIF prompts played a significant role in driving positive affective attitudes and behavior change and were consistently perceived as a low-burden, engaging form of prompt that encouraged walking across participants.
Salience messages	Factorial: no significant effects on affective constructs; MRT: no significant proximal effect on daily affective reflection and anticipated affect	Despite the lack of statistical significance, participants noted that salience messages helped them reframe walking as something to look forward to rather than an obligation.	Salience messages may support changes in affective attitudes through repeated reflection, although the effects were not statistically detected; longer exposure or further optimization may be needed.
Planning prompts and reminders	Factorial: no significant effects on affective constructs	Despite the lack of statistical significance, participants found the planning prompts useful for reflecting on ways to make walking more enjoyable and incorporating it into their daily routine.	Planning messages may enhance positive affective attitude over time by encouraging reflective strategies, although the effects may require a longer intervention window to manifest.

^a^MRT: microrandomized trial.

Collectively, these results highlight the potential of GIF walking suggestions to support behavior change and foster intrinsic motivation for PA. These findings also build on and extend previous evaluative conditioning studies conducted in controlled laboratory environments. While previous research has demonstrated that pairing PA cues with positively valenced stimuli can improve implicit attitudes and affective evaluations [36-38], such studies have typically been brief and conducted with young adult populations in highly controlled settings. In contrast, WalkToJoy applied this mechanism in a real-world mHealth context among middle-aged and older adults. Compared to HeartPhone [39], a similar mHealth intervention that passively presented exercise imagery on smartphone lock screens, WalkToJoy delivered emotionally engaging stimuli as part of an active behavioral cue—delivering GIF images alongside walking suggestions via SMS text message or Multimedia Messaging Service. This design more directly integrated affective elicitation with the behavioral prompt itself, potentially allowing for a stronger affective-behavioral link and the influencing of behavior.

This final point about how WalkToJoy implemented affective conditioning may also offer an explanation for why we saw large increases in steps for this factor but not in impact on affective attitudes and valuations. Affective associations take time to shift, and given our sample size and the 6-week study duration, we likely did not have enough time to detect a statistically significant impact of a single component on affective associations. However, given that the GIF images were embedded in walking suggestions, they may have had a more immediate impact in that they made participants more receptive to the walking suggestions themselves, resulting in more near-term activity. The interview findings we presented offer support for this conjecture that increased receptivity to walking suggestions may have played a key role in mediating the effects of this intervention.

The MRT analysis revealed no significant increase in a 4-hour step count immediately following the delivery of GIF versus text-only prompts (*P*=.55). This null finding may be due to design-related factors, including the potential misalignment between the analysis window and the suggestive—not directive—nature of the message framing in the walking suggestions. Affective processes—the primary mechanisms of change in this intervention—are known to evolve gradually and accumulate over time. As such, the lack of immediate impact on the short-term proximal outcome may reflect this slower trajectory. In addition, participants noted in the interviews that GIF prompts were interpreted as flexible, day-long reminders rather than immediate calls to action. This perception appeared to stem from the suggestive and nonurgent wording of the prompts (eg, *Could you go for a short walk this afternoon?*), which allowed participants to incorporate walking into their routines on their own terms. By reducing immediate pressure, the framing used in WalkToJoy seemed to align with participants’ preferences, fostering positive engagement with the intervention while supporting their ability to make choices about when to walk.

This observation highlights the nuanced role of message framing in health interventions [40,41,43], emphasizing that both directive and suggestive framings have distinct advantages and limitations depending on the context and user group. Directive messages, which emphasize immediate action (eg, *Take a short walk now to feel energized!*), may be better suited for individuals requiring a clear prompt to overcome inertia or decision fatigue as they provide explicit guidance and can create a sense of urgency. Such framing has been shown to be effective in other interventions such as HeartSteps, where more assertive prompts successfully encouraged immediate walking [75]. However, directive framing can also risk inducing psychological resistance, particularly in individuals who value autonomy and flexibility. In contrast, suggestive, nondirective framing—as used in this study—offers a more autonomy-supportive approach. By presenting walking as an open-ended opportunity rather than an immediate demand, this type of framing may reduce pressure, increase perceived control, and encourage participants to act in ways that align with their routines and preferences. This aligns with self-determination theory, which posits that behaviors motivated by autonomy and perceived choice are more likely to be internalized and sustained over time [76]. However, the trade-off is that this framing might not elicit immediate behavioral responses, as observed in the lack of significant proximal step count increases in this study. Future mHealth interventions could benefit from leveraging both approaches, tailoring message framing to the specific goals of the intervention and the needs of diverse user groups. For instance, directive prompts could be used strategically in moments requiring immediate action, whereas suggestive prompts could support longer-term habit building and user autonomy.

Our analyses found no significant impacts of salience messages and planning prompts on affective and behavioral outcomes, suggesting that these components may require refinement or that larger or longer studies would be needed to demonstrate impact given that affective processes take time to evolve. Despite the lack of statistical significance, qualitative data suggest that salience messages facilitated meaningful attitudinal shifts by reframing walking as an enriching and mindful activity. Through the emphasis on the positive aspects of walking via message framing [40,41,43], participants felt that salience messages transformed walking from an obligation into an intrinsically rewarding experience. Similarly, planning prompts, though not statistically significant, were highly valued by participants for their role in enhancing the enjoyment and personalization of walking. These observations are consistent with previous research underscoring the importance of personalization in enhancing user engagement and satisfaction in health interventions [77,78].

In contrast to the more automatic mechanism of evaluative conditioning used in the GIF walking suggestions, the salience and planning components of WalkToJoy aimed to support belief updating, which is theorized to be slower and more reflective in nature. Previous interventions have used message framing or action planning to promote walking among older adults [41,51-53], but few have explicitly targeted affective attitudes as a mechanism of change. WalkToJoy advances this approach by using positive framing and planning prompts to elicit emotionally positive walking experiences and then foster reflection on what these experiences mean for how participants feel about walking. Thus, these components slowly nudged participants to revise how they thought they felt about PA using their own experiences of evidence that walking may be something that they actually like. Our qualitative findings indicate that this process unfolded for at least some of our participants, providing initial evidence for its promise.

### Limitations

This study, as a proof-of-concept investigation, faced several inherent limitations. First, this study was not fully powered for the factorial experiment, limiting the ability to detect smaller or more nuanced effects of the individual intervention components. In addition, the study’s duration of 6 weeks was relatively short, precluding the ability to assess WalkToJoy’s long-term impact on affective mechanisms and behavior change.

Another notable limitation is the potential sampling bias of requiring participants to own a Fitbit, which may have resulted in a more health-literate and self-motivated sample than the general population. However, this was consistent with the intervention’s target audience—individuals who are interested in becoming more active but face barriers to maintaining motivation—making this sample appropriate for evaluating the intervention’s initial promise. Participants were generally moderately active and reported favorable attitudes toward walking at baseline, potentially limiting observable improvements but also suggesting higher receptivity to the intervention. Future work should examine how such interventions perform among individuals with more negative affective associations with PA and lower baseline activity levels.

### Conclusions

This proof-of-concept study of the WalkToJoy intervention demonstrates the feasibility and potential of affective-based strategies for promoting PA. The smile-inducing walking suggestions in particular played a significant role in enhancing engagement and motivation, underscoring the value of affect-driven and visually engaging approaches as a fatigue-resistant intervention strategy. While salience and planning prompts showed promise, further refinement and extended deployments may be necessary to optimize their effects. These findings highlight the importance of integrating affective dimensions into digital health interventions to foster sustained engagement and meaningful health behavior change.

## Appendix

Multimedia Appendix 1 Semistructured interview protocol.

Multimedia Appendix 2 Demographic characteristics of the exit interview participants.

## Abbreviations

ART: affective-reflective theory
GEE: generalized estimating equations
mHealth: mobile health
MRT: microrandomized trial
PA: physical activity

## Data Availability

The datasets generated or analyzed during this study are not publicly available due to privacy and confidentiality agreements with participants. However, deidentified data may be made available from the corresponding author on reasonable request.
