# Supplementary material for: Improving Affective Associations With Physical Activity via a Message-Based mHealth Intervention (WalkToJoy): Proof-of-Concept Study
Source: J Med Internet Res. 2025 Aug 8;27:e75792. doi: 10.2196/75792 (PMC12374139; doi:10.2196/75792)
Supplement: Multimedia Appendix 1 [file jmir_v27i1e75792_app1.docx]

**Multimedia Appendix**

**A1. Semi-structured Interview Protocol**

*Integration to daily life*

- During the study, what did your schedule look like on a daily basis (How busy were you on average)?
- How did your schedule affect your ability to go for a walk?
- How did walking fit into your daily life or routine?
  - When did you usually go for a walk?
  - How did walking impact your mood or mindset throughout the day?
  - Over the course of the 6-weeks, did your walks change in any way? (e.g., timing, duration or intensity of the walks)
    - Did the walks feel any different compared to before the study?
    - Did your enjoyment of walking change in any way?
    - Did you find the way you fit your walks into your daily routine different in any way?
    - How sustainable do you feel your current walking routine is?
- Reflecting on the study period, how do you feel about the way you incorporated walks into your daily routine?

*General Experience with WalkToJoy study*

- Overall, how was your experience with the WalkToJoy study, meaning receiving messages everyday and having to complete surveys daily and weekly?
- What did you find valuable about WalkToJoy? What was not so valuable?
- Throughout the study, you were encouraged to reflect on your walks, your feelings towards those walks and their positive benefits.
  - Can you think of instances where the reflections change your walking behavior and attitude towards walking?
  - In general, what did you focus on when reflecting on your walks?
  - How did the reflections make you feel?

*Barriers to Walking*

- During the study, can you think of any barriers you experienced when trying to go for a walk? (e.g. weather, mood, schedule, social situations, etc.)
  - How often did you experience these barriers?
  - How did these barriers make you feel?
  - How did these barriers affect your future walks?
  - Did any part of the intervention help you deal with those barriers during the study?
  - Are any of these barriers going to be a problem going forward? What are they, and why?

*Experience with GIF & Non-GIF messages*

- What did you think of the prompts to go for a walk?
  - Were you able to notice the prompts when you received them? Can you recall any prompt that stood out to you? (provide examples of the prompt)
  - How often did you go for a walk as a result of the prompts? What was that experience like?
  - Do you ever think the prompts influenced your walking behavior later, even if you didn’t act on it right away?
  - How did the prompt make you feel, and did that change your walks in any way?
  - Was there ever a time where you got the message and wanted to go for a walk but were unable to? If so, what were the barriers?

*GIF only*

- What did you think of the GIFs that were sent along with the prompts to go for a walk?
  - How did you feel when you saw the GIFs?
  - Do you have any favorite GIFs that you can remember?
  - How did you feel when you didn’t receive the GIFs?
  - How do you think the GIFs affected your walks if at all?

*Salience only*

- You received messages on things to pay attention to during future walks everyday at 12PM. *(provide examples of the prompt)*
  - Were you able to notice the messages?
  - Can you recall any of them? Can you recall any that you particularly liked or found helpful?
  - How did you feel when you received these messages?
  - Did you think about the content of the message throughout your day and when going for walks? How did the message impact your walks?
- Was there ever a time the message you got seemed inappropriate? (i.e., not matching your mood or situation) How did that make you feel?

*Planning only*

- You were asked to create, update or continue with the same strategy to enhance your walking experience every Monday.
  - Were you able to remember your strategy throughout the week?
  - Can you remember any strategy that you thought were particularly helpful? Any that turned out not to be helpful?
  - Did you try these strategies for your walks?
- In what ways did your strategies impact your walks, if at all?
  - How did the planning make you feel towards walking?
- How did you feel when you were asked to continue with the same strategy vs. when you were asked to try something new?
  - Were there any situations where you wanted to change your strategy or stay with the same when you were prompted to do otherwise?
    - If so, how did you deal with this situation (e.g., modify slightly or entered the same strategy)?
- You were reminded of your strategies twice a week. Did you find them helpful in remembering your strategy? Or did they feel redundant?
  - How did you feel when you received them?

*Message Timing*

- What did you think about the timing of the prompt messages you received?
  - Did the timing of the messages feel appropriate for you?
  - Can you think of any situation where the messages felt out of place in terms of timing (e.g., should be sent in the evening instead of afternoon; received while working)?
  - Can you think of any situation where the messages came at just the right time?
- Hypothetical
  - If you could modify the timing of the messages, when would you receive them?
  - If you could snooze a message to be sent at a later time, would you use this feature? In what ways do you think it will be helpful?
  - If you could collect messages and GIFs that you like (similar to bookmarks or favorites on web browsers), would you use them? In what ways do you think it will be helpful?

*Impact on walking behavior and attitude*

- Now that you have completed the study, how would you describe your relationship with walking?
  - How would you describe your relationship with physical activity/exercise in general?
- How do you identify yourself in relation to exercise (walking)?
  - Did this identity change throughout the course of the study?

*Closing*

- Is there anything else you'd like to tell us that we haven't already covered?
- Do you have any questions for us?
