# Supplementary material for: Improving Affective Associations With Physical Activity via a Message-Based mHealth Intervention (WalkToJoy): Proof-of-Concept Study
Source: J Med Internet Res. 2025 Aug 8;27:e75792. doi: 10.2196/75792 (PMC12374139; doi:10.2196/75792)
Supplement: Multimedia Appendix 2 [file jmir_v27i1e75792_app2.docx]

**A2. Demographic characteristics of the exit interview participants**

| Participant | Intervention group  (GIF-Salience-Planning) | Age range | Gender |
| --- | --- | --- | --- |
| P3 | F-T-F | 40 – 49 years | Woman |
| P5 | T-F-F | 40 – 49 years | Woman |
| P6 | T-F-T | 50 – 59 years | Woman |
| P7 | T-T-F | 40 – 49 years | Man |
| P12 | F-T-T | 40 – 49 years | Man |
| P24 | T-T-T | 60 – 69 years | Woman |
| P32 | T-T-T | 40 – 49 years | Woman |
| P33 | F-F-F | 40 – 49 years | Man |
| P42 | F-F-T | 60 – 69 years | Man |
